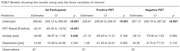# The Relationship Between Amyloid Burden, Cognition and Neuropsychiatric Symptoms in Individuals with Amnestic Cognitive Decline

**DOI:** 10.1002/alz70857_102118

**Published:** 2025-12-25

**Authors:** Alia Alokley, Juan‐Camilo Vargas‐González, Hessah A Alotibi, Nico Paulo Dimal, Martin Ingelsson, David F. Tang‐Wai, Carmela Tartaglia

**Affiliations:** ^1^ Memory Clinic, Toronto Western Hospital, University Health Network, Toronto, ON, Canada; ^2^ Toronto Dementia Research Alliance, Toronto, ON, Canada; ^3^ Tanz Centre for Research in Neurodegenerative Disease, University of Toronto, Toronto, ON, Canada; ^4^ Krembil Brain Institute, University Health Network, Toronto, ON, Canada; ^5^ Canadian Concussion Centre, Krembil Brain Institute, University Health Network, Toronto, ON, Canada

## Abstract

**Background:**

Depression and anxiety are frequently observed neuropsychiatric symptoms in amnestic cognitive decline. However, the effect of anxiety and depression on cognition in amnestic patients with and without amyloid positivity [amyloid β‐ (Aβ)], a key biomarker in Alzheimer's disease, has not been thoroughly examined. We evaluated the relationship between Aβ positivity and anxiety and depression on cognitive scores in a cohort of patients with amnestic mild cognitive impairment.

**Method:**

Participants with amnestic cognitive impairment who underwent Positron Emission Tomography (PET) amyloid as part of their assessment at the University Health Network Memory Clinic were included. Anxiety and depression were assessed using the Neuropsychiatric Inventory Questionnaire (NPI‐Q). Cognition was assessed using the Toronto Cognitive Assessment (TorCA) total score. We categorized people as normal or abnormal based on age‐specific normative data. Patients with a non‐amnestic syndrome were excluded from the study. We used a TOBIT regression to evaluate the effect of anxiety and depression on cognition in patients who were either PET amyloid positive or negative

**Result:**

A total of 67 patients were included in the study: 37 with PET amyloid‐positive and 30 with PET amyloid‐negative results. There was no significant difference in age (PET positive mean age = 70.9 +/‐ 8.4 SD; PET negative mean age=69.3 +/‐ 6.5 SD; *p* = 0.37) or sex (24M;13F PET amyloid‐positive; 18M;12F PET amyloid‐negative; *p* = 0.81) among the two groups. Using a TOBIT model regression, the presence of anxiety negatively affects overall cognition scores with a *p*‐value of 0.042. Although not significant, there appears to be a greater effect of anxiety in those who are PET amyloid‐negative (positive *p* = 0.166; negative *p* = 0.06). Additionally, we found that the presence of depression is not significantly associated with changes in cognitive scores; *p* = 0.34

**Conclusion:**

Anxiety contributes to cognitive decline in amnestic individuals, regardless of whether they have PET amyloid‐positive or PET amyloid‐negative scans. Although only a trend, anxiety may have more impact in those who are PET‐amyloid negative.